# Development of an Evidence-Informed Solution to Emotional Distress in Public Safety Personnel and Healthcare Workers: The Social Support, Tracking Distress, Education, and Discussion CommunitY (STEADY) Program

**DOI:** 10.3390/healthcare10091777

**Published:** 2022-09-15

**Authors:** Janet Ellis, Melissa B. Korman

**Affiliations:** 1 Department of Evaluative Clinical Sciences, Sunnybrook Research Institute, Toronto, ON M4N 3M5, Canada; 2 Department of Psychiatry, Sunnybrook Health Sciences Centre, Toronto, ON M4N 3M5, Canada; 3 Institute of Medical Sciences, University of Toronto, Toronto, ON M5T 2S8, Canada

**Keywords:** healthcare worker occupational safety, job satisfaction, hospitals, occupational health, organizational development

## Abstract

Public safety personnel (PSP) and healthcare workers (HCWs) are frequently exposed to traumatic events and experience an increased rate of adverse mental health outcomes compared to the public. Some organizations have implemented wellness programming to mitigate this issue. To our knowledge, no programs were developed collaboratively by researchers and knowledge users considering knowledge translation and implementation science frameworks to include all evidence-informed elements of posttraumatic stress *prevention.* The Social Support, Tracking Distress, Education, and Discussion Community (STEADY) Program was developed to fill this gap. It includes (1) peer partnering; (2) distress tracking; (3) psychoeducation; (4) peer support groups and voluntary psychological debriefing following critical incidents; (5) community-building activities. This paper reports on the narrative literature review that framed the development of the STEADY framework and introduces its key elements. If successful, STEADY has the potential to improve the mental well-being of PSP and HCWs across Canada and internationally.

## 1. Introduction

Public safety personnel (PSP) and healthcare workers (HCWs) are exposed to potentially traumatic events in the line of duty, including routine exposure to human suffering. This puts them at high risk for negative mental health outcomes such as posttraumatic stress injury/disorder, compassion fatigue, and burnout [1,2,3,4,5,6,7]. This paper reports on the findings of a narrative literature review that framed the development of a novel wellness program for public safety personnel (PSP) and healthcare workers (HCWs), and details the components of the program. We will use the term posttraumatic stress injury (PTSI) in this paper, in keeping with public safety and other federal programs that have adopted the term posttraumatic stress “injury” rather than “disorder”, due to potential negative connotations associated with the term “disorder” [8].

## 2. Narrative Review

### 2.1. Mental Health Needs of Public Safety Personnel (PSP)

After the attacks on the World Trade Centre on 11 September 2001, a surge of research revealed that many emergency responders and civilians exposed to the traumatic event later suffered from mental health conditions including PTSI, panic disorder, depression, increased substance use, and dissatisfaction with life [9,10,11]. This showcased the impact on PSP of being exposed to potentially traumatic events in their daily work, from terrorist attacks to natural disasters to accidental injury [12,13,14].

**Figure 1:**
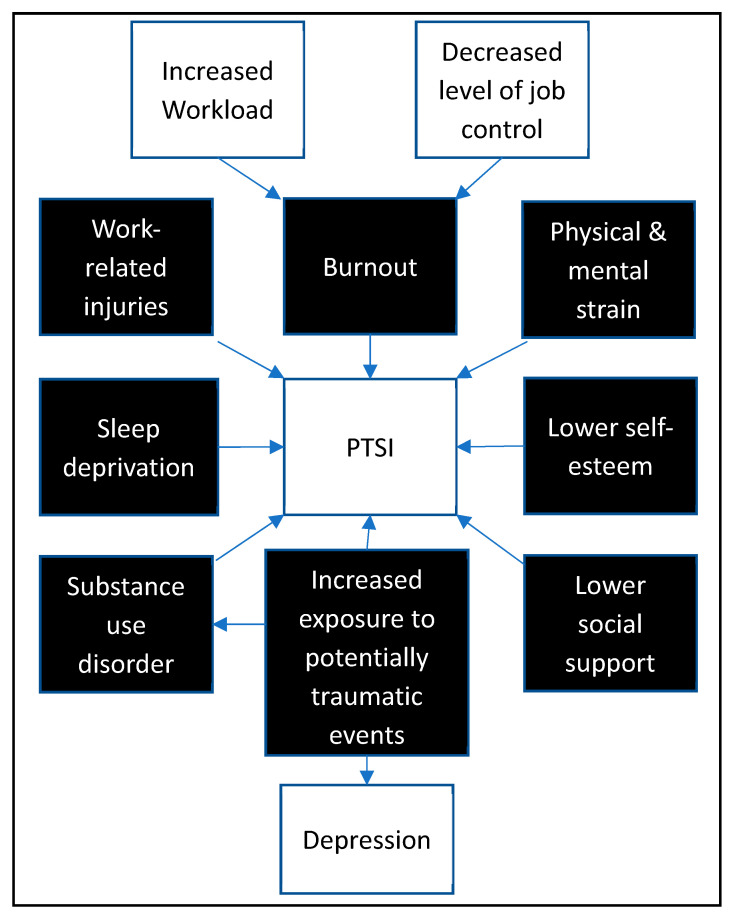
Relationship between adverse physical and mental health in public safety personnel as reported in the literature.

PSP are under high pressure to perform and are at risk of occupational stress injuries, including anxiety, depression, substance use, sleep deprivation, burnout and PTSI (Figure 1) [1,2,3,9,10,11,12,13,14,15,16,17,18,19]. A 2018 report of a survey of 5813 Canadian PSP revealed that 26.7% suffered from symptom clusters of more than one mental disorder [20]. The researchers concluded that data suggest substantial mental health difficulties for PSP across Canada, highlighting the need for population-specific research and solutions. In another study, 27.8% of Canadian PSP reported thoughts of suicide and 4.6% had attempted suicide [21]. Job-specific research has revealed elevated rates of PTSI in multiple PSP roles; up to 37% of firefighters, up to 44% of police officers, and up to 22% of paramedics, compared to up to 9% in the general public [20,22,23,24]. A study of 112 firefighters revealed that 58% displayed binge-drinking behaviours [1] and 59% were sleep-deprived. Due to the dangerous work environment, such issues in this population could be catastrophic.

PSP are also at risk for compassion fatigue, as their occupations include routinely helping traumatized and suffering individuals [25]. Compassion fatigue is the emotional and physical burden often felt by individuals helping those in distress, which results in a reduced capacity to feel empathy [26]. People often do not realize that they are suffering from compassion fatigue [27], yet compassion fatigue can affect all areas of life, from personal relationships with partners and friends to parenting and their ability to perform a job with humanity [28]. Compassion fatigue can contribute to an increased risk of PTSI and burnout [27], which can be defined as a “psychological syndrome in response to chronic interpersonal stressors on the job.” Burnout is composed of three dimensions: emotional exhaustion, cynicism or depersonalization, and a sense of reduced efficacy or accomplishment [29], and has been associated with physiological changes in the brain and the body [30,31].

### 2.2. Mental Health Needs of Healthcare Workers (HCW)

Burnout and compassion fatigue are endemic occupational stress injuries in healthcare which lead to increased turnover and absenteeism, reduced job satisfaction and quality of care, and strained relationships [6,21,32]. HCWs show a greater propensity for job absenteeism due to illness or disability than other work sections [6]. A 2003 study revealed that 45% of Canadian physicians report advanced states of burnout [33]; this longstanding issue has only been exacerbated by the COVID-19 pandemic [34,35,36]. Depression, PTSI, substance use, and suicide are also more common in HCWs than in the general population, with an even higher risk following adverse events, critical incidents, and disasters (e.g., pandemics) [4,7,32,37,38,39,40,41,42,43,44,45,46,47,48]. In one study, 33% of Canadian nurses reported thoughts of suicide, and 8% had attempted suicide. There is a need for ongoing programming to support positive mental health in HCWs, prevent adverse outcomes, and intervene with those suffering from psychological distress, in order to reduce the risk of long-term suffering and disability, and the negative impact this has on the system at-large.

### 2.3. Barriers to Mental Healthcare in PSP and HCW Populations

Unfortunately, PSP and HCWs share both the heightened risk of adverse mental health outcomes. These groups also experience similar barriers to accessing mental health support. Organizational programming, including employee assistance programs, often provide reactive interventions, where staff are required to identify their own distress and ask for support. These resources are often under-utilized, due, in part, to the negative stigma associated with help-seeking [49]. The stigma of mental health disorders includes perceived weakness in character, with possible feelings of status loss and discrimination [50]. Self-stigma is a significant barrier to treatment in HCW and PSP populations, as seen in a case described by Royle, Keenan, and Farrell (2009) where a police officer refused critical incident stress debriefing after witnessing a traumatic crime scene of a child murder, due to fear of crying in front of his colleagues. The officer admitted that he thought of his fellow colleagues who had previously sought help as “weak” and undeserving of the uniform. He was frustrated to think that others could be speaking of him similarly. Many police officers report not attending psychological services due to fear of being seen by other officers or their supervisors and being placed on administrative duties [38]. Higher stigma both decreases one’s likelihood of accessing support due to stigma and has been associated with a higher likelihood of alcohol abuse, posttraumatic stress, and depression [51,52,53,54]. In the first responder populations, there have been efforts to decrease stigma regarding help-seeking and the need to appear “tough” with acknowledgement of the need to address the emotional demands of the job and recognition of PTSI as a workplace injury; unfortunately, limited change has been reported to the historically strong culture of negative stigma and John Wayne syndrome.

John Wayne syndrome describes the tendency to distance from emotions in order to cope with the difficulty of the work-life of a first responder, and in response to the fear of loss of status. This can lead to cynicism and emotional withdrawal in all facets of life [55,56]. John Wayne syndrome has been well reported in PSP and has been shown to impact staff and learners [3,16,56], with a similar culture seen in HCWs [57,58]. Both populations are at high risk of negative mental health outcomes due to similar occupational stressors of exposure to human suffering in high-pressure situations, along with expectations that they are the “heroes”. This has been exacerbated by the COVID-19 pandemic [59,60].

### 2.4. Evidence-Informed Elements of Occupational Stress Injury Prevention

Many concepts, strategies, and/or techniques have been described in the literature as mechanisms for addressing and/or mitigating negative mental health outcomes in work groups, including HCW and PSP populations. The benefits of key factors or “elements of the solution” consistently reported are described here.

#### 2.4.1. Social Support

While self-stigma and John Wayne syndrome might prevent a PSP or HCW from turning to family, friends, or coworkers for support, multiple studies have shown that perceived social support is a protective factor for PTSI, particularly for those who experienced more severe trauma [61,62]. Increased social support, job resources (including job control), and rewards were correlated with reduced burnout in PSP; coworker support diminished the harmful impact of emotional demands [63]. Similarly, encouragement to discuss rather than suppress thoughts might be helpful to decrease posttraumatic stress symptomatology [64]. Due to the aforementioned barriers to accessing mental health resources, i.e., stigma and John Wayne syndrome, PSP and HCWs may not feel comfortable turning to coworkers for support without prompts or encouragement to do so. Based on the major potential benefit, it is evident that social support should be encouraged for individuals who are at risk of developing PTSI and included in any wellness programming for these vulnerable populations.

Peer support groups can be used to encourage conversation between colleagues, or any individuals with shared lived experiences, in order to increase individuals’ perception of peer support while also reducing stigma and countering cultural norms which encourage the repression of distress. The peer support model is preferred over other mental health resources by many HCWs [48,65,66]. This method of support can be particularly useful when implementing a new program in cultures where individuals are wary of outsiders, as it leverages pre-existing trusted work relationships to increase program acceptance while decreasing the time needed to build trust between supporters and staff [48,65,66,67].

#### 2.4.2. Leadership Engagement to Reduce Stigma

Leadership engagement, along with psychoeducation, can be used to reduce the negative stigma associated with expressing emotional distress and help-seeking behaviours in work environments [58]. Teaching leaders and frontline workers about normal stress reactions, as opposed to pathologizing them, might help to increase comfort with a discussion of emotional reactions and knowing when to access mental health services. Further, if leaders can model vulnerability, staff might feel more comfortable expressing their own distress, or at least worry less about fear of repercussions (i.e., being placed on administrative duties) in response to reporting distress [49,50]. In fact, positive leadership and unit cohesion were reported by military personnel to reduce the perception of mental health stigma and barriers to care [68]; this concept could be applied to other PSP and HCW populations. Suggested interventions include veteran workers encouraging new recruits to attend mental health services to challenge stigma [50] while reinforcing leadership skills and promoting higher levels of unit cohesion [68].

#### 2.4.3. PTSI Prevention Strategies

Primary prevention of PTSI occurs before exposure to a potentially traumatic event. The PSP literature suggests that increasing preparedness and awareness pre-exposure can help to mitigate the negative psychological impact of traumatic events [69]. This can include increasing individual resilience and/or identifying those with inherent risk factors; one mechanism that can be used to accomplish this is psychoeducation.

Many techniques and strategies can be taught to support individuals to increase their resilience. This could include general stress-reduction exercises (such as tactical breathing, visualization, and SMART goal setting), reflective practice, self-compassion and positive self-talk, or techniques drawn from mindfulness-based stress reduction or other brief therapies, such as acceptance and commitment therapy [70,71,72,73]. In addition to resilience building, psychoeducation has been used to mitigate distress by increasing awareness of common stress reactions, individual vulnerability to PTSI and other negative outcomes, and describing coping styles that reduce the risk of developing PTSI.

A study on police respondents to 9/11 revealed that social support can play a significant role in preventing PTSI, although it may be less significant in recovery for those who already suffer from PTSI [10]. Thus, increasing perceived social support is a vital part of primary prevention. It is important to note that social support goes beyond the occupational environment; social support from friends and family has been shown to be important to an individual’s wellbeing and quality of life, increasing one’s ability to cope with traumatic events, and acting as a protective factor from PTSI [61,62,74,75]. When coping with disaster, individuals report the highest levels of social support coming from their families [76].

Secondary prevention includes early intervention after the traumatic event, with the aim of preventing PTSI. Secondary prevention usually occurs within one-month post-trauma; some interventions occur within the first week post-trauma. For example, critical incident stress debriefing, as part of the critical incident stress management program is a one-off, small group, crisis-focused discussion that occurs at least 24 h post-incident [77,78]. Though there is mixed evidence for the utilization of critical incident stress debriefing in PTSI prevention [79,80,81], when used properly (e.g., as part of a program) it has been shown to increase the quality of life and posttraumatic growth and to reduce PTSI and substance use in PSP populations [82,83,84]. Debriefing has also been shown to benefit HCWs [85].

Identification of those in distress enables timely intervention, reduces mental/physical sequelae and improves productivity [3,86,87,88]. Furthermore, this can protect compromised individuals from being exposed to another traumatic event prior to receiving adequate emotional support. Multiple screening tools have been created and validated for the purposes of detecting individuals suffering from psychological distress and symptoms of PTSI. Tools developed to assess the severity of posttraumatic stress symptoms such as the Posttraumatic Stress Disorder Checklist-Civilian Version [89], the Impact of Event Scale-Revised [90], and the Posttraumatic Stress Disorder Symptom Scale-Self-Report Version [91] are often used as screening tools. Although diagnosis should not be made by self-report screening tools alone, a 2005 systematic review revealed over 85% mean diagnostic efficiency for screening tools [92]. Screening as a form of secondary prevention should also include questions regarding risk factors for PTSI and other adverse mental health outcomes. For instance, PSP who do not have families or perceived family support may be at increased risk of PTSI; therefore, questions might be included regarding the perception of social support at work and home. This is not a static risk factor and therefore should not only be measured before a traumatic event; emerging PTSI may impact close relationships and decrease one’s sense of perceived social support.

Tertiary prevention is providing interventions for someone who has already developed PTSI and sequelae. Interventions could include psychoeducation, support, and providing access to specialized help or evidence-based management of PTSI (e.g., medication, cognitive processing therapy, prolonged exposure therapy, eye movement desensitization, and reprocessing therapy). Psychoeducation as tertiary prevention would include providing information about PTSI and its treatments to instill hope in patients while increasing their sense of control and knowledge regarding possible courses of treatment.

### 2.5. Existing Mental Health Programs for PSP/HCW Populations

Many programs have been devised to support the mental health needs of PSP and HCW [58,78,93,94,95,96,97,98,99]. Mental wellness programs increase the ability to cope with cumulative occupational stress and can improve health outcomes for HCWs and PSP [58]. Existing wellness programs are limited in scope, flexibility, and duration, have not yet demonstrated sustainability or long-term effectiveness, were not developed using knowledge translation or implementation science approaches, and/or do not include all evidence-based components of “the solution,” i.e., all potential mediators of psychological resilience and decreased occupational stress injury reported in the literature. Many existing occupational stress injury programs take a reactive approach, treating mental illness rather than focusing on prevention. We set to develop a new wellness program to address this gap, building on identified strengths and weaknesses from existing programs. A brief summary of existing programs identified during the narrative review and a comparison to the novel program are included below.

### 2.6. Summary

PSP and HCW populations face a heightened risk of occupational stress injuries due to the nature of their work, along with practical and culture-related barriers to accessing necessary support. The literature outlines numerous ways to prevent and treat said occupational stress injuries. The current report describes the development of a wellness program that leverages existing evidence to offer a comprehensive set of supports that can meet the needs of diverse work groups with varying needs.

## 3. The STEADY Program

The primary aim of the STEADY program was to mitigate PTSI in PSP and HCWs using primary, secondary, and tertiary prevention strategies. Secondary aims were to:

- Mitigate the barriers to accessing support by bringing resources to the target audience in an acceptable format and encouraging culture change;
- Include resources that can suit the needs of diverse groups;
- Highlight program flexibility so that it can be adapted to target audiences (and therefore can be highly deployable and scalable).

**Figure 2:**
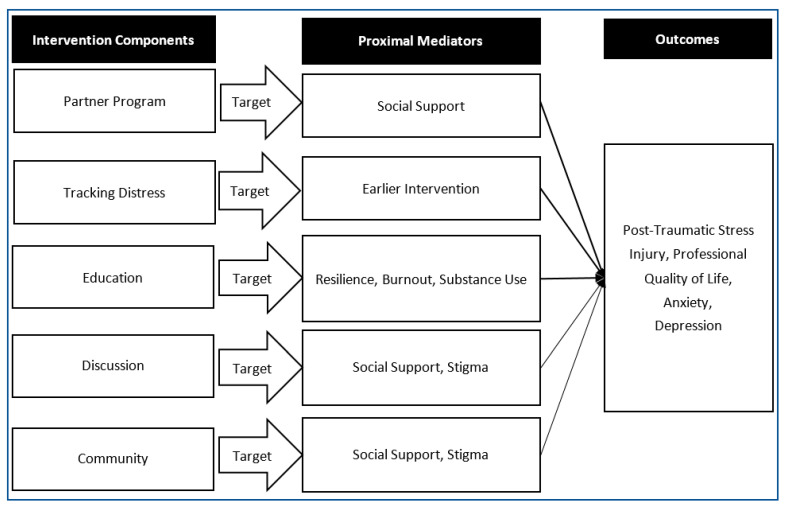
Hypothesized mediation of intervention effect for each “Element of the Solution” selected for inclusion in STEADY based on literature review and consultation with stakeholders and experts.

The Knowledge to Action Framework [100] and Consolidated Framework for Implementation Research [101] were selected to guide program development and plans for implementation. Narrative review and stakeholder meetings were conducted to broaden our understanding of the mental health challenges associated with HCW and PSP work, evidence-backed approaches to tackling these issues, and successes and failures of past programming (including a thorough review of programs available, what they include, whether they have been systematically implemented and evaluated and all pertinent reported outcomes). STEADY was named based on the five main components that create the pillars for the program’s framework: social support (through peer partnering), tracking distress for proactive intervention (rather than distress-initiated intervention), education regarding the problem to normalize distress and introducing strategies to build personal resilience, discussion (through peer support groups and voluntary debriefing following critical incidents), and community-building initiatives. These interventions/approaches were identified during the narrative review and selected based on their potential to impact proximal mediators of the outcomes of interest. In this case, we chose to focus on mitigating negative outcomes of PTSI, anxiety, and depression and improving the positive outcome of professional quality of life (which includes components of compassion fatigue and burnout mitigation). Figure 2 outlines the hypothesized mediation of effect for each intervention/approach included in STEADY.

### 3.1. Key Components

**Peer partnering** was selected primarily as a means of increasing a sense of social support, as it has been shown to protect PSP and HSW against negative outcomes of facing traumatic events [102,103]. This element of STEADY leverages pre-existing, trusted work relationships to increase program acceptance and decreases the time needed to build trust between supporters and staff, similar to a peer support model [48,65,66]. Peer partnering can be facilitated by unit leaders or identified STEADY champions; staff might choose a partner with a similar schedule, or can buddy up and check in at the beginning and end of each shift, or after a critical incident. Partners should provide mutual emotional and social support and encourage each other to practice self-care. STEADY also promotes social support through the education, discussion and community elements (described below).

**Tracking Distress** using monthly, bimonthly, or quarterly wellness assessments (based on group needs and capacity) would allow program facilitators to identify those in need of support. Wellness assessments were designed to be completed either in online or in paper formats, and included the following validated questionnaires:

- Beck Depression Inventory—Second Edition (BDI-II) [104];
- PTSD CheckList for DSM-5 [105];
- Single Item Burnout Scale [106];
- CAGEAID (to evaluate Substance Use) [107];
- Generalized Anxiety Disorder 7-Item Questionnaire [108];
- Multidimensional Scale of Perceived Social Support [74].

We developed an algorithm of responses based on the severity of screening scores. Responses would be provided to participants with a summary of results (i.e., whether the individual scored positively for any outcome of interest), trends in scores (i.e., whether scores have increased or decreased since the last submission), and suggested resources or strategies based on their distress profile. Strategies might include ways to increase self-care, reflective practice, mindfulness exercise, and other appropriate techniques. Resources might include links to self-help applications, helpful articles, or even organizational resources (e.g., supports offered through the employee assistance program). Where possible, personalization would be included; for example, when individuals report sleep disturbances on the BDI-II, they could be provided with a list of resources/suggestions for improving the quality of sleep.

As safety is a serious consideration in distress screening, disclaimers were included in wellness assessments noting that the screen is not a substitute for mental health care. STEADY includes a suicide prevention intervention with a stepwise escalation protocol effective in the general population, following the Stanley and Brown crisis intervention [109]. Where scores are increasingly or consistently high, staff are offered a phone call with a qualified member of the STEADY Team (JE), along with usual resources, and encouraged to contact their family doctor or resources offered through the employee assistance program. Response to high scores or active suicidal ideation also includes crisis line information and/or reminders to go to the nearest emergency department.

**Education workshops** were designed to be virtual or in-person. They cover a range of mental health and wellness topics, such as understanding and overcoming burnout and compassion fatigue, distinguishing normal vs. acute and posttraumatic stress reactions, skills for coping and resilience-building, meditation and mindfulness, having difficult conversations with patients, and self-care. The goal in developing these workshops was to normalize work stress, increase awareness of personal vulnerability and risk factors for adverse mental health outcomes, teach skills for bolstering resilience and self-care and reducing stress and mitigate the impacts of John Wayne syndrome and the negative stigma associated with seeking support. Workshops would ideally be conducted face-to-face by trained facilitators or champions from target organizations who can engage with the group.

**Discussion** is characterized by formal peer support groups or informal check-ins and voluntary critical incident debriefing. Peer discussion provides a safe space for learning, building trust, and normalizing stress [64,110,111]. Though biweekly hour-long sessions are recommended, the frequency and length of peer support groups, as part of the STEADY program, would vary according to the target population based on factors associated with the work environment (e.g., scheduling demands).

Voluntary critical incident stress debriefing is included to increase resilience and reduce PTSI [88,112,113,114]. Debriefs are recommended to occur from 24 h to one week following the potentially traumatic event. As “critical incidents” may be defined and experienced differently by individuals or units of PSP and HCWs, staff are asked to request debriefs after any experience they define to be a “critical incident” or particularly stressful and traumatic event. Debriefs are structured conversations between facilitator(s) and individuals who felt impacted by the critical incident (other leadership or staff that were not impacted are asked not to attend). Details of the event and personal accounts and feelings are discussed, along with psychoeducation regarding trauma and ways to self-care, and the importance of caring for oneself. Conversations do not include operational critique or “what went wrong,” rather details of what occurred and what it felt like are described by participants. Throughout the conversation, facilitators reinforce the fact that the event is over.

**Community** is promoted by STEADY both at work and at home. The STEADY framework includes the option for the family to participate in workshops and other programming, where deemed appropriate by the target audience, to help family members understand the demands of the job and to encourage open conversation around distress at home to increase the overall sense of social support. At work, active leadership engagement plays a critical role in engaging teams and encouraging culture change. A sense of community promotes supportive discussion, reduces stigma, and increases help-seeking behaviours. STEADY promotes culture change to achieve an atmosphere conducive to support without judgement, as increasing mutual support and decreasing stigma will increase organizational resilience.

### 3.2. What Does STEADY Add?

The framework for the STEADY program was created considering the evidence-based components of existing international PTSI prevention programs, as well as critical gaps identified and reported weaknesses of said interventions (Table 1) [78,93,94,95,96,97,98,99]. The following list of existing programs is non-exhaustive but highlights the main programs for which information was readily available at the time of STEADY program development and identified during the narrative review. Many additional wellness programs have been developed, implemented and/or reported on in the target populations in response to the pressures of the COVID-19 pandemic [115,116,117,118,119,120]. Still, we are unaware of equally comprehensive programs developed based on the best evidence for occupational stress injury prevention in PSP/HCW populations using strategies from the field of knowledge translation and implementation science.

**Critical incident stress management (CISM)** promotes coping and provides support for staff after disaster deployment [77,78]. There are seven components; 1. pre-crisis preparation; 2. demobilization and staff consult; 3. defusing; 4. critical incident stress debriefing (CISD); 5. individual crisis intervention; 6. family CISM; 7. follow-up/referral [77]. Apart from pre-crisis preparation, the components are reactive rather than proactive [77,78]. CISM has been shown to increase self-help, self-efficacy, and community resilience after potentially traumatic events, as well as reduce burnout, absenteeism, PTSI, and substance use [121]. CISD encourages discussing traumatic events in small-group, crisis-focused discussions [77]. CISD is controversial as the literature is inconsistent. This may be because many studies used CISD alone (despite its recommended use as part of a broader, more comprehensive program), or earlier than recommended (at least 1-day post-incident). CISD has been shown to increase QOL and posttraumatic growth and reduce symptoms of posttraumatic stress injury and substance use in public safety personnel [83,122,123,124] and has been reported by nurses to reduce stress [125]. STEADY adds peer partnering and drop-in peer support groups to promote social support, as well as online resources, to the CISM intervention.

**Wounded Warriors Canada Before Operational Stress (BOS)** focuses on resilience, helping individuals to take charge of their own mental health and the effects of occupational stress [93]. BOS is psychoeducation-based, running over 8 weeks (16 h total), followed by 10 months of follow-up [93]. BOS provides a safe place for PSP to discuss work stress and symptoms, but not necessarily with colleagues from their organization [94]. Evaluations of BOS in PSP populations have revealed decreased PTSI symptoms, as well as improvements in QOL, stigma, and perceived social support [126]. Similar to BOS, STEADY includes drop-in peer support groups to discuss work stress and education that promote resilience, but STEADY does so within the PSP organizations, to develop a long-term work community of support and culture change. BOS is limited in the number of participants (up to 10 PSP at a time) and needs a clinical psychologist to run the program; STEADY is flexible, on-site, ongoing and can be peer-led.

**Couples Overcoming PTSI Everyday (COPE)** helps families to learn and move forward from PTSI, as it is associated with poor familial adjustment, reduced intimacy, and higher divorce rates [95,97]. COPE starts with a 5-day knowledge-focused intervention with five couples with similar experiences [95]. Coaching continues for 6 months (3 sessions per month), to maintain knowledge [95]. STEADY provides education and support to PSP separate from their loved ones as well as concurrently and creates a community of individuals with similar experiences. STEADY adds a long-term supportive community within units/organizations and focuses on prevention as well as intervention.

**Canadian Forces Return to Work program** helps injured individuals to return to work in a timely fashion, for effective rehabilitation, mitigating loss of skills and social connections [94]. It includes enhanced communication, flexible work schedules with modified duties, and a supportive work environment to set the individual up for success [94]. STEADY does not include a return to work program but it does encourage enhanced communication and a supportive work environment, with peer partners to remind each other that it is okay to take an operational pause when needed. STEADY adds ongoing, long-term structured resources, with a focus on both prevention and early intervention.

**The Working Mind First Responders (TWMFR**), formerly the Road to Mental Readiness (R2MR) program was developed by the Canadian Armed Forces to reduce mental health stigma and increase resilience and access to care [96]. TWMFR includes educational programs for primary staff, leadership, and trainers [96]. Primary staff receive a 4 h session on stigma and barriers to care, the “Big 4” coping and resilience skills, practical scenarios, videos of lived experiences, and the Mental Health Continuum Model self-assessment tool [96,99]. A meta-analysis revealed that TWMFR increased resilience skills and reduced the stigma of mental illness, with the use of learned skills at follow-up; resilience skills were partially maintained at 3 months and only 14% of respondents reported feeling able to support someone else’s mental health [99]. Limitations included high attrition rates and short-term follow-up; more research is needed to evaluate long-term effects and determine the impact of TWMFR on mental health status [99]. STEADY includes similar elements in workshops, and adds ongoing education, discussion of traumatic events, distress tracking, and encourages ongoing social support and a sense of community.

**The Mental Agility and Psychological Strength (MAPS)** program for primary prevention of PTSI consists of four modules with psychoeducation about wellbeing and PTSI, practical skills and relaxation and mindfulness training [98]. MAPS encourages social support, normalizes stress reactions and bolsters coping, self-efficacy and self-care. The MAPS program was evaluated on firefighters in western Australia; delivered in 4 weekly 1 h group sessions by a psychologist [98]. Despite a low attrition rate, there was no evidence for primary prevention of PTSI or impact on social support or coping strategies [98]. STEADY and MAPS are both evidence-informed, with a similar focus on psychoeducation and social support. STEADY is long-term, adds debriefing, distress tracking and online resources, and includes several strategies to increase social support.

**Hand-n-Hand Peer Support** participants are triaged and then matched with a peer support volunteer and have the option to request to be paired with an individual in the same discipline, specialty, and/or level of seniority. There is also the option for group peer support. Participants may attend webinars or join small group discussions on COVID-19 and mental health support. This initiative launched in March 2020, so service evaluation remains in the preliminary stages. STEADY adds distress tracking and family social support and is offered in the staff’s work environment.

Many existing interventions do not include elements of family social support, routine tracking of distress, or highlighting building a sense of community while being proactive and long-term. For instance, The Working Mind First Responders (TWMFR) and The Mental Agility and Psychological Strength (MAPS) programs focus primarily on psychoeducation but do not track individual distress or offer ongoing opportunities for discussion, as STEADY does. Additionally, Couples Overcoming PTSI Everyday (COPE) and Hand-n-Hand Peer Support are both distress-initiated and off-site, whereas STEADY is proactive, engaging individuals in their own work environment before a problem arises, thereby increasing accessibility and focusing on prevention as well as early intervention. Further, the multiple elements of STEADY can be adapted and selected to fit target units’ needs; e.g., some groups might prefer informal walkabout check-ins for the “discussion” element, rather than formal drop-in groups. We encourage adapting the method of program delivery according to unit-specific needs, e.g., offering hybrid in-person + virtual programming. To our knowledge, no other program includes options for adaptation to individual contexts.

Though this paper does not report on the evaluation of STEADY, it is important to note the gaps in research regarding the effectiveness of existing programs in order to contextualize the need for this novel program. Despite some evidence of positive outcomes from existing programs for PSP/HCW populations, systematic reviews on the topic have described significant heterogeneity across studies, noted that improvements in outcomes of interest were small and short-term, and limited strength of evidence in many reports due to the failure to control for pre-existing PTSIs [127,128]. Di Nota et al. (2021) concluded that “extant literature is unclear regarding the long-term effectiveness of various coping strategies employed by PSP following exposure to work-related potentially psychologically traumatic events”. Further, research generally focuses on individual-level, rather than organization-level, outcomes. This is likely related to the interventional focus on the short-term well-being of the individual, rather than the long-term status of an organization. STEADY is unique in that it targets both the individual and the group with the aim of establishing long-term proactive supports. It includes a variety of supportive options that any individual can access, and also influences organizational culture by bringing the intervention to the worksite, encouraging a sense of community, and striving to decrease the stigma associated with emotional distress and accessing support. Reports on the evaluation of STEADY, considering both the impact on individuals and organizational culture/outcomes, will follow.

## 4. Next Steps and Implications for Practice

STEADY was developed to be deployable and scalable, utilizing knowledge translation and implementation science frameworks. It takes an on-site approach (i.e., being conducted within the work environment), highlights leadership engagement to model vulnerability, and prioritizes developing a sense of community in order to encourage uptake and acceptability within target populations that are notoriously unaccepting of emotional support resources for a variety of reasons. Nonetheless, gaps between theory and practice have been documented across disciplines [129,130] and many evidence-based, theory-driven interventions do not withstand the test of time [131]. Therefore, the crucial next steps for the STEADY program include evaluating the feasibility, sustainability, and effectiveness in practical settings, using a knowledge translation and implementation science lens and considering organization-level (as well as individual-level) outcomes.

As is evident in the bidirectional Knowledge-to-Action Cycle, knowledge translation (including translation of evidence-based programs into practice) is an iterative, bidirectional process. Initial trials of program feasibility have been conducted (data analysis is underway; results will follow). Characteristics and needs of target populations (i.e., workgroups where STEADY has been implemented) influence the way that STEADY is implemented in each area, in keeping with our guiding Knowledge to Action Framework. Findings regarding the implementation process and associated outcomes continuously inform program adaptation. As STEADY evolves to increase acceptability and scalability, the potential benefit of this program grows.

If we can successfully achieve the goals of program development and efficiently disseminate our findings for large-scale deployment, the STEADY program has the potential to create more resilient PSP and HCWs worldwide. Not only would this improve the well-being of the individual program participants but could have secondary effects, such as improved organizational wellness and better outcomes for patients and their families. The ongoing STEADY projects will inform future interventions (by creating data relevant to improving STEADY and the implementation of any wellness program in the target populations), and research to prevent suffering, disability, and even death by suicide in HCWs and PSP.

## Figures and Tables

**Table 1:** Comparing elements of STEADY with other occupational stress programs for healthcare workers and public safety personnel.

Program	Peer Social Support	Routine Tracking of Distress	Psycho- Education	Defusing/Debriefing Discussions	Online Resources/Community	Ongoing	Within One’s Own Work Environment	Proactive, Not Distress Initiated
**STEADY**	**✓**	**✓**	**✓**	**✓**	**✓**	**✓**	**✓**	**✓**
Critical Incident Stress Management	✓	X	✓	✓	X	✓	✓	X/✓
Before Operational Stress	✓	X	✓	X	X	X (1 year)	X	✓
Couples Overcoming PTSI Everyday	✓	✓	✓	✓	X	X (6 months)	X	X
Return to Work	✓	✓	✓	X	X	X	✓	X
The Working Mind—First Responders	✓	X (self)	✓	X	X	X	X/✓	✓
Mental Agility and Psychological Strength	✓	X	✓	X	X	X	✓	✓
RESPECT	✓	X	✓	x	X	X	✓	✓
Recognize, Evaluate, Advocate, Coordinate, and Track (REACT)	✓	X	✓	x	X	X	✓	✓
Resilience@work (R@w)	X	X	✓	X	✓	X	✓	✓
Resilience in Stressful Events	✓	X	X	✓	X	✓	✓	X
Reflective Listening, Assessment, Prioritization, Intervention and Disposition	X	X	✓	X	✓	X	✓	✓

## Data Availability

Not Applicable.
